# Melt-Mixed Thermoplastic Nanocomposite Containing Carbon Nanotubes and Titanium Dioxide for Flame Retardancy Applications

**DOI:** 10.3390/polym11071204

**Published:** 2019-07-19

**Authors:** C. Cabello-Alvarado, P. Reyes-Rodríguez, M. Andrade-Guel, G. Cadenas-Pliego, M. Pérez-Alvarez, V.J. Cruz-Delgado, L. Melo-López, Z.V. Quiñones-Jurado, C.A. Ávila-Orta

**Affiliations:** 1 CONACYT-Consorcio de Investigación y de Innovación del Estado de Tlaxcala, C.P. 90000 Tlaxcala, Mexico; 2 Centro de Investigación en Química Aplicada, Saltillo, 25315 Coahuila, Mexico; 3 CONACYT-Instituto Mexicano del Petróleo, Eje Central Lázaro Cárdenas Norte 152, 07730 Ciudad de Mexico, Mexico; 4 CONACYT-Unidad de Materiales, Centro de Investigación Científica de Yucatán, A.C., Mérida C.P. 97205, Yucatán, Mexico; 5 Innovación y Desarrollo en Materiales Avanzados A.C., Grupo POLYnnova, Carr. San Luis Potosí-Guadalajara 1510, Nivel 3, Local 12, Lomas del Tecnológico, San Luis Potosí S.L.P. C.P. 78211 Mexico, Mexico

**Keywords:** nanocomposite, polypropylene, carbon nanotube, titanium dioxide

## Abstract

The study of polymeric nanocomposites is a possible alternative to conventional flame retardants. The aim of the present work is to investigate the effects of carbon-nanotubes (CNT) and TiO_2_ nanoparticles (NPs) on the thermo-mechanical, flammability, and electrical properties of polypropylene (PP). In this work, PP-TiO_2_/CNT nanocomposites were obtained with TiO_2_/CNT mixtures (ratio 1:2) through the melt extrusion process, with different weight percentage of nanoparticles (1, 5, and 10 wt %). The PP-TiO_2_/CNT nanocomposites were characterized by DSC, TGA, MFI, FTIR, XRD, and SEM. It was possible to determine that the thermal stability of the PP increases when increasing the content of NPs. A contrary situation is observed in the degree of crystallinity and thermo-oxidative degradation, which decreased with respect to pure PP. The TiO_2_ NPs undergo coalition and increase their size at a lower viscosity of the nanocomposite (1 and 5 wt %). The mechanical properties decreased slightly, however, the Young’s modulus presented an improvement of 10% as well as electrical conductivity, this behavior was noted in nanocomposites of 10 wt % of NPs. Flammability properties were measured with a cone calorimeter, and a reduction in the peak heat release rate was observed in nanocomposites with contents of nanoparticles of 5 and 10 wt %

## 1. Introduction

Avoiding or preventing fires helps decrease injuries or human losses in automotive industry [1]. Different causes arise for these types of events, such as mechanical problems, electrical failures, or car accidents, since most automotive liquids are flammable. The automotive parts manufactured with different polymers are flammable and must be protected against fires [2]. For these materials, some flame retardants are applied only in the exterior, and some carbonaceous and ceramic films help to improve their flame-retardant effect [3,4,5,6]. 

One of the most common polymers used to obtain polymeric nanocomposites is polypropylene (PP), which is a semi-crystalline thermoplastic widely used in applications such as textile, films, bottles production, and piping, and is also the most used in the automotive industry [7,8,9]. However, PP has some limitations, such as being sensitive to heat and oxidative degradation, as well as its fragility. This polymer by itself is prone to crack or vulnerable to mechanical failures and, like most polymers, has low electrical and thermal conductivity; for these reasons, the addition of nanoparticles or nanocharges has been adopted with the purpose of changing properties of neat polymer [10,11]. 

The incorporation of nanoparticles to improve polymers properties depends in great extent on wt % of nanocharge used. A clear example of this effect was observed in ethylene vinyl acetate (EVA) nanocomposites with different nanoclay content (1–15 wt %). Thermal stabilization was achieved when the nanoclay content was between 2.5–10 wt %. Outside this range, the EVA thermal stability was not significant. In general, polymers properties are improved with low percentages in weight of nanocharge (<3 wt %), however, several researchers have studied high nanocharges percentages [12,13,14]. The flame-retardant effect of carbon nanotubes (CNT) has been researched in epoxy, polystyrene, polyaniline, polypropylene, and polyurethane polymeric matrixes, and the polymer/CNT nanocomposites are effective in producing continuous structured network [15,16]. A significant reduction in the peak heat release rate was observed in PP/CNT nanocomposites that contain 0.5–4.0 wt % of CNT [17].

Antunes et al. obtained PP and carbon nanofibers (CNF) nanocomposites by melting extrusion with electrical properties, and, taking into account the percolation threshold, formation of this nanocomposite showed similar conduction of Hall effect at CNF concentrations of 5%. At higher CNF contents, no significant improvements were achieved since tunnels conduction decreased in the extent polymer crystallinity increased [18]. The PP/CNT nanocomposites improve their electrical properties when increasing CNT content; the electrical percolation threshold was reached at CNT content of 1 and 2 wt % [19].

The addition of inorganic nanoparticles in polymeric matrix and the combination of different types of particles are used for obtaining multifunctional materials. The nanoparticles such as TiO_2_, SiO_2_, CaCO_3_, ZnO, Ag, and nanoclays help to improve the physical and mechanical properties as well as flame retardant activity, thermal stability, oxidation, and permeability, among others [20,21]. The titanium dioxide (TiO_2_) nanoparticles are ceramic materials widely used thanks to their properties as reinforcement for polymeric materials due to their long-term stability. Aydemir et al. [22] report the production of PP/TiO_2_ nanocomposites, where mechanical properties as stress resistance and elasticity module were widely favored with the addition of TiO_2_ in the polymeric matrix. Esthappan et al. [23] report the production of PP-TiO_2_ nanocomposites for use like fibers, since addition of TiO_2_ nanoparticles improve thermal stability of polypropylene, besides improving polymer crystallinity.

There are different methods for polymeric nanocomposites synthesis. Among them is found the melting extrusion method, which favors homogeneous dispersion of nanocharges, and is considered eco-friendly and economically viable for the industry, since it does not require the use of solvents and can yield high production volumes [24,25]. 

This research work studies the synthesis and evaluation of nanocomposites of polypropylene with TiO_2_ and CNT nanoparticles blend. The nanocomposites were obtained by melt extrusion method. The purpose of using these additives was to improve the properties of thermal stability, electrical conductivity, and flame retardant of PP.

## 2. Materials and Methods

### 2.1. Material

MWNTC were provided by Cheap Tubes Inc. and have an average diameter from 20 to 40 nm, length of 10–30 µm, and purity of ≥ 90% wt. The TiO_2_ nanoparticles were provided by DuPont, with a particle size from approximately 200 nm and semispherical morphology rutile phase. The polymeric matrix PP with fluid index of 0.76 g/10 min. supplied by Polímeros Nacionales (México, México) was used.

### 2.2. Methods

#### Synthesis of PP/CNT Nanocomposites

**Figure 1:**
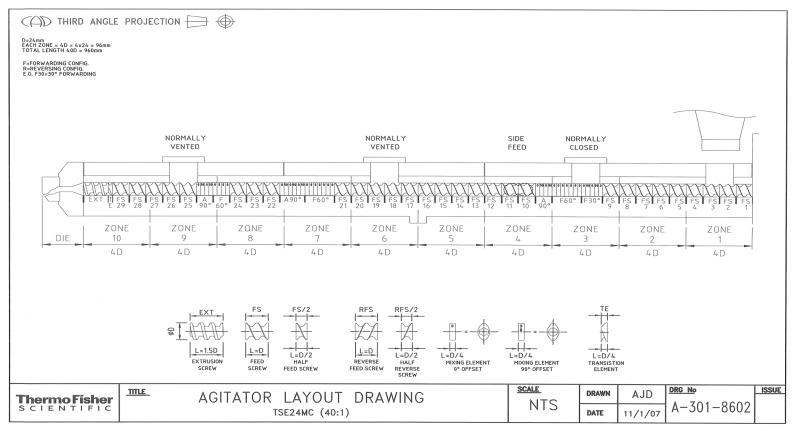
Configuration for the extrusion process.

The preparation of PP nanocomposites with a mixture of TiO_2_/CNT was performed by the melting extrusion process. The masterbatch preparation of PP/TiO_2_/CNT was conducted in a twin-screw extruder lab size from Thermo Scientific model Prism TSE-24MC, screw diameter of 24 mm, L/D ratio 40:1, and temperature profile of 180 °C and rotational speed of 100 rpm. Low shear strengths were used to improve particles dispersion in polymeric matrix in the screw configuration, which is shown in Figure 1. 

There were prepared three nanocomposites of PP (1, 5, 10 wt %) with different weight percentages of CNT and titanium dioxide (TiO_2_) nanoparticles. Table 1 lists the amounts used. The nanocomposites are identified as PP-TiO_2_/CNT-X, where X means the weight percentage of nanocharges (CNT+ TiO_2_) and PP without particles. It was decided to use these percentages due to some authors reporting improvements in the physical and chemical properties of polymers when adding CNT [26].

### 2.3. Characterization

#### 2.3.1. X-Ray Diffraction (XRD)

For nanocomposites structural analysis, wide angle X-ray diffraction technique (WAXD) was used, conducted in diffractometer from Siemens model D-5000, operating at current intensity of 25 mA and voltage of 35 kV, to obtain Cu Kα radiation with a wavelength equivalent to 1.54056 Å.

#### 2.3.2. Fourier Transform Infrared Spectroscopy FTIR (ATR)

For FT-IR analysis a Thermo Nicolet infrared spectrometer, model MAGNA 550 (GMI, Minneapolis, Minnesota, USA), was used. The conditions at which these analyses were performed are the following: scanner 100, resolution of 16 cm^−1^, and wave interval from 4000 to 500 cm^−1^ with ATR support. 

#### 2.3.3. Melt Flow Index (MFI)

The melt flow index was obtained using Dynisco plastometer, which consists of heating a barrel to melt material. Aw piston press was loaded with melted material to make it flow through die with a circular orifice of 2.1 mm of diameter and length of 8 mm. This test was performed under ASTM D1238-40 standard.

#### 2.3.4. Thermal Stability (DSC and TGA)

For evaluating thermal properties, thermogravimetric analysis was used. The equipment used was Dupont Instruments model 951 (TA Instruments, New Castle, Pennsylvania, USA), operated at heating rate of 10 °C/min in nitrogen atmosphere with gas flow of 50 mL/min. The approximate weight of samples was of 10 mg and was analyzed in the interval of temperature from 25 to 800 °C. The Differential Scan Calorimetry was carried out under ASTM D3418 standard and thermal analyzer Q2000 from TA Instruments (New Castle, Pennsylvania, USA), with standard cell.

The degree of crystallinity (Xc) was calculated using with the following equation:

(1) $$X_{c}\left(\%\right)=\left[\frac{\Delta H_{f}}{\left(1-\emptyset \right)\Delta H^{*}}\right]\times \left(100\right)$$

where Δ*H_f_* is the fusion heat or formation of PP in the nanocomposites, ΔH* is the formation heat of PP with crystallinity of 100% equivalent to 209 J/g and Ø is the fraction of weight of TiO_2_ and CNT [27]. 

#### 2.3.5. Scanning Electron Microscopy (SEM) 

For the determination of size and morphology for each one of the components, a JOEL Field Emission Scanning Electron Microscope model JSM-7401F (JEOL, Peabody, MA, USA) was used. The microscope acceleration voltage was of 3.0 kV using the LEI secondary electrons detector. 

#### 2.3.6. Mechanical Properties Analysis

For measuring mechanical properties, tension tests were performed on model 4301 Instron universal machine (Instron corporation, Norwood, Massachusetts, USA), at 5 mm min^−1^, with different lengthening percentages (0, 60, 400, and 700%).

#### 2.3.7. Electrical Resistivity 

These evaluations were made three times. For electrical resistivity, thickness plates were used, and they were covered in both sides with silver paint. The device used was Keysight LCR [inductance (L), capacitance (C), and resistance (R)] meter model E 4980 A, over 20 Hz to 2 MHz and LCR meter model ZM2372, from 0.001 Hz to 100 kHz. The electrical conductivity was calculated using the Equation (2) [28]:

(2) $$\sigma =\frac{1}{\rho }$$

where σ is the electrical conductivity and ρ is the electrical resistivity.

#### 2.3.8. Calorimetric Cone

For evaluation of combustion properties of nanocomposites, a calorimetric cone from Fire Testing Technology was used following the method described under ASTM E1354 standard. The evaluation of samples was made in horizontal position and the position of heat flow generator cone was also in horizontal position. The samples measurements were from 100 mm × 100 mm × 3 mm and were obtained by compression molding. The calorimetric cone was calibrated at 5 kW with methane flow, the flow in the extraction duct was of 24 L/s, and the analyzer was calibrated with 20.95% of oxygen. Heat flow for assessing samples was 35 kW/m^2^. The sample was placed in aluminum paper tray with same dimension of sample and 1 cm height, leaving the surface to evaluate with free area of 100 mm^2^. This was placed in the sample holder adjusting distance between cone and surface of sample to 25 mm.

## 3. Results and Discussion

### 3.1. X-ray Diffraction

**Figure 2:**
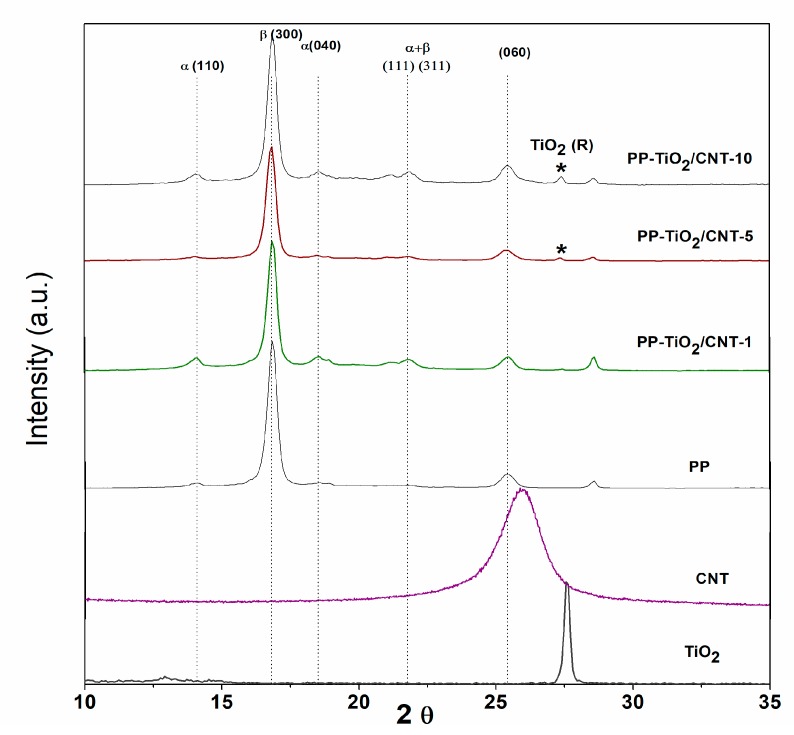
X-ray diffraction patterns of nanocomposite PP-TiO_2_/CNT (1,5,10%), polypropylene (PP), carbon-nanotubes (CNT) and TiO_2_.

In Figure 2, there are shown PP, and CNT and TiO_2_ nanoparticles XRD diffractograms with the aim to compare diffraction patterns of base materials with nanocomposites of PP-TiO_2_/CNT. The diffractograms of samples with PP showed signals located at 2Ɵ angles of 14.1, 16.1, 16.8, 21.5 and 25.5°, which correspond to planes (110), (300), (040), (111), and (060) of PP crystalline phase, and the other signal located at 2Ɵ angle of 21.1° can be correlated to phases α(111) and β(311) of PP. In the nanocomposites, signals characteristic of CNT [29.1° plane (100)] were not detected, since polypropylene signals are superimposed. The nanocomposites containing TiO_2_ showed the characteristic signal located at 28.5° (110) [29,30].

The crystallinity can be more affected in the PP-TiO_2_/CNT-10 nanocomposite where change related to polymer crystallinity is detected, increasing the nanocomposites signals. In 2013, Wang et al. studied PP nanocomposites with carboxylate nucleating agent (NTC) and the XRD diffractograms showed a signal at 2Ɵ of 16.51°, which corresponds to crystalline plane (300) of β-hexagonal crystalline phase, showing that NTC has clear effect of nucleation in PP [31]. Similar results were obtained by Zohrevand et al., in 2014 when studying PP/TiO_2_ nanocomposites with 1, 3, and 5 vol%. The intensity of this β phase is significant only in nanocomposites with 1 vol%, while with 3 and 5 vol%, the intensity of peak is not significant. Besides this, they report that presence of a peak in 2Ɵ at 21.1° can be correlated to alpha and beta phase, and these results confirm that presence of TiO_2_ nanoparticles induces β-form crystal formation in PP [32]. 

### 3.2. Fourier Transform Infrared FTIR (ATR)

**Figure 3:**
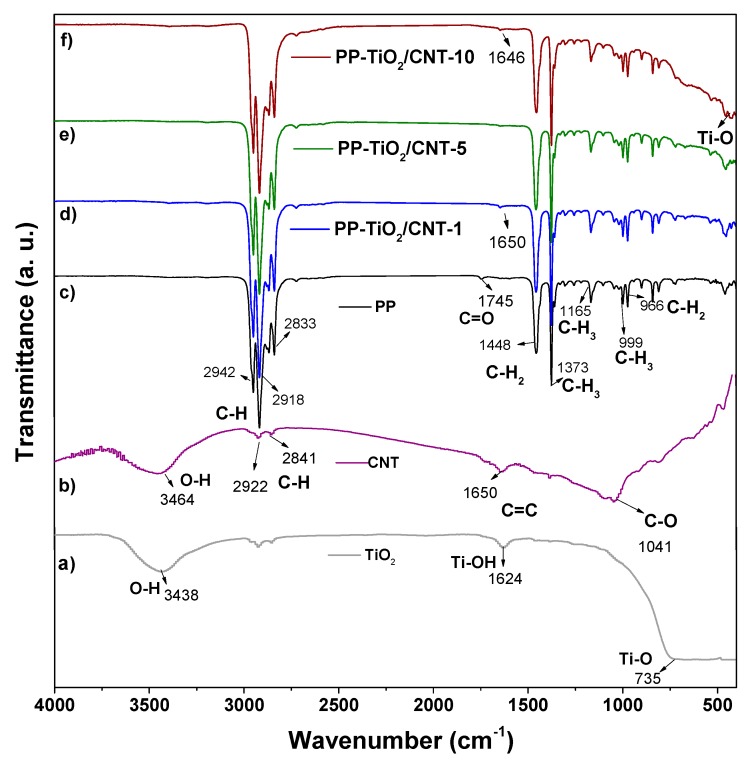
FT-IR spectra of nanocomposite PP-TiO_2_/CNT (1, 5 and 10%), polypropylene (PP), carbon nanotubes (CNT) and TiO_2_.

The spectroscopy results of FT-IR are shown in Figure 3, where it can be noted that nanocomposites (Figure 3d–f) show transmittance signals characteristic of PP located in the range from 3000 to 2800 cm^−1^, which correspond to asymmetric and symmetric C-H stretching vibration of methylene (CH_2_) and methyl (CH_3_) groups. Signals corresponding to flexions of CH_2_ and CH_3_ bonds are localized in 1448 cm^−1^ and 1373 cm^−1^, respectively. The FT-IR spectrum of TiO_2_ nanoparticles (Figure 3a) shows three signals: in 3438 cm^−1^ corresponding to hydroxyl group O-H, in 1624 cm^−1^ of Ti-OH, and at 735 cm^−1^ corresponding to Ti-O bond [33,34]. The FT-IR spectrum of CNT is shown in Figure 3b, and the signal of stretching of O-H bonds was detected at 3464 cm^−1^, which existing in CNT. Also, in 2918 and 2841 cm^−1^, signals of C-H bond were detected, and in 1650 cm^−1^ there were detected signals from stretching of C=C vibrations and at 1041 cm^−1^ of C-O bond [35,36].

The nanocomposites of PP with contents of 5 and 10 wt % (Figure 3e,f), showed signals corresponding to PP and TiO_2_ resin, and in 443 cm^−1^ a signal that corresponds to Ti-O bond was detected. The other signal that increases intensity in the nanocomposites with greater charge percentages (5 and 10%) is the one corresponding to CH_3_, with values in the order of 1373 cm^−1^. 

This coincides with results obtained by Hashing et al. in 2004, when preparing nanocomposites with PP and CNT by mechanical pulverization, where they obtained variation in the intensities of the signals at 1373 cm^−1^, attributing this result to that some PP chains are strongly bonded to CNT walls, in consequence to strong actions of cut, compression, and friction of mixing [37].

The FT-IR spectrum of neat PP processed at same conditions as nanocomposites shows a small signal in 1745 cm^−1^, which is related to its thermo-oxidative degradation and the formation of carbonyl groups. The presence of these carbonyl groups into range of 1810 to 1660 cm^−1^ [38] is reported in the literature. On the other hand, the FT-IR spectra nanocomposites do not show the signal in 1745 cm^−1^ which suggests that the NPs mix of CNT and TiO_2_ inhibits or reduces the thermo-oxidative degradation effects during processing of PP.

In this regard, it has been reported that the use of TiO_2_ as an additive reduces the thermal degradation of PP. When there is an increase of TiO_2_ concentration, the signals attributed to asymmetric and symmetric C-H stretching vibration (3000–2800 cm^−1^), exhibit an intensity rise, which can suggest an increase in thermal stability of PP. The absence of bands in the wave number range 3600–3200 cm^−1^ indicates that formation of hydroperoxides was not favored [39].

### 3.3. Evaluation of Melt Flow Index (MFI) 

The results of the evaluation of melt flow index (MFI) for different samples are listed in Table 2. The MFI values suggest that the addition of nanoparticles increase the viscosity, as a result of the materials becoming less fluid after increasing the CNT and TiO_2_ content. The PP-TiO_2_/CNT-10 nanocomposite showed greater viscosity increase (0.24 MFI g/10 min), in comparison with the value shown by PP without charge (0.76 g/10 min). The nanocharges have a high aspect ratio, which favors strong intermolecular interaction with the polymeric matrix; the adsorption of PP chains in the surface of nanocharges increases viscosity. These interactions increase the deformation resistance to hinder polymer flow in melted state, thereby making difficult or limiting the polymer flow in melted state [40].

### 3.4. Thermogravimetric Analysis (TGA)

**Figure 4:**
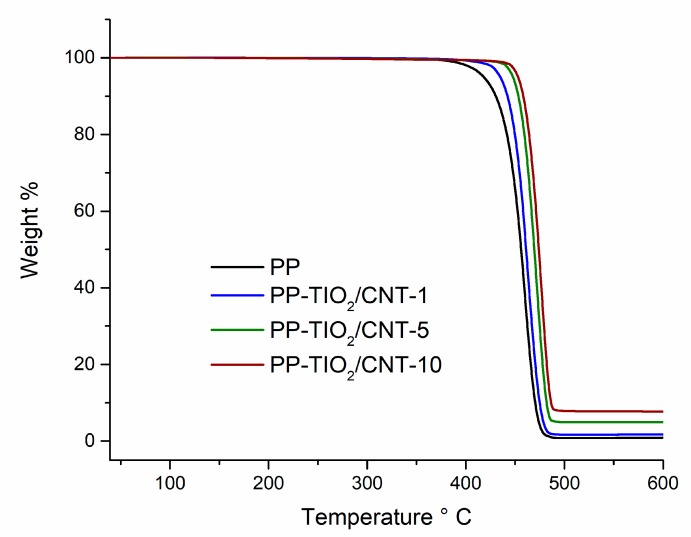
Thermogravimetric analysis of polypropylene (PP) and nanocomposites PP-TiO_2_/CNT (carbon nanotubes).

To define thermal stability of nanocomposites, thermogravimetric analysis was performed. Figure 4 shows these results and it can be noted that materials have similar thermal behavior; the samples analyzed do not exhibited weight loss related to water adsorbed in surface materials. The first weight loss is in the range 358–500 °C, this is attributed to breaking of chains existing in polypropylene structure [41].

The nanocomposites thermograms show that mass losses at 5 wt % and 50 wt % are detected at different temperatures in each nanocomposite (Table 3). In general, the temperatures T_5%_ and T_50%_ increased with the charges content in the nanocomposites. The PP-TiO_2_/CNT-10 nanocomposite showed the highest temperature compared to nanocomposites with less nanocharge content. The 5 wt % decompositions for neat PP in PP-TiO_2_/CNT-1, PP-TiO_2_/CNT-5, and PP-TiO_2_/CNT-10 occurred at 435, 448, and 454 °C, respectively. This means that the addition of charges increases the decomposition temperature in 18, 31, and 37 °C. Similar behavior was shown when the nanocomposites reached a 50 wt % of decomposition. 

These results indicated that using TiO_2_ and CNT blend improves PP thermal stability; therefore, these combinations are a viable alternative to obtain flame retardant materials. Similar results have been reported when using TiO_2_ and CNT nanoparticles individually in polypropylene [42,43].

To define a comparison of maximum decomposition temperature (T_max_) the second derivative was used in TGA analysis. The values obtained in several formulations corroborate the difference of thermal stability between nanocomposites and PP without charge. The nanocomposites showed greater T_max_, the PP-TiO_2_/CNT-10 sample increased its T_max_ in 16 °C above pure PP. 

The increase in starting decomposition temperature can be attributed to the increase in the addition strength in the PP interface and the CNT and TiO_2_ nanoparticles. When proper interface interaction exists, the particles are able to restrain the movement of polymer chain, making it more difficult that the breaking of the polymer chains occurs at lower temperature. In consequence, the degradation temperature of nanocomposite is shifted to a higher temperature [44].

Other element to consider when improving thermal stability of nanocomposites is the transport barrier effect of the mass of the CNT hollow structure, and these structures can trap the free radicals generated during PP thermal [45,46]. 

### 3.5. Differential Scan Calorimetry (DSC) Analysis

**Figure 5:**
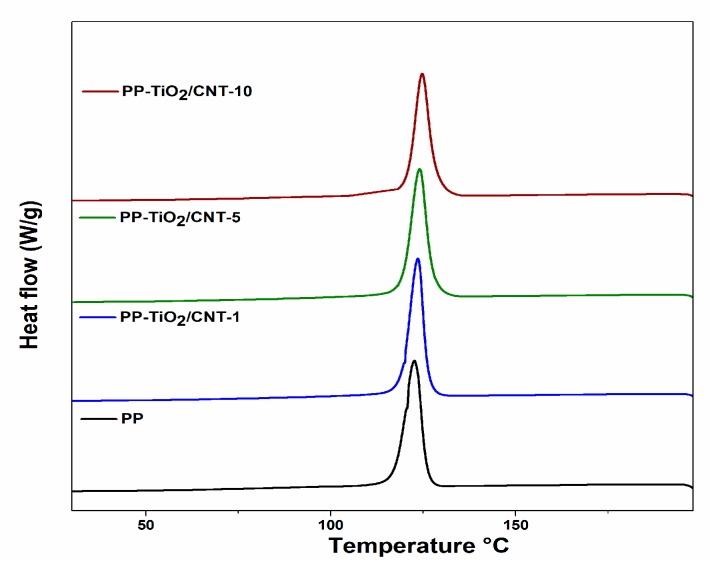
Differential scanning calorimetry (DSC) crystallization exotherms of pure polypropylene (PP) and PP-TiO_2_/CNT (carbon nanotubes) (1, 5, 10%).

**Figure 6:**
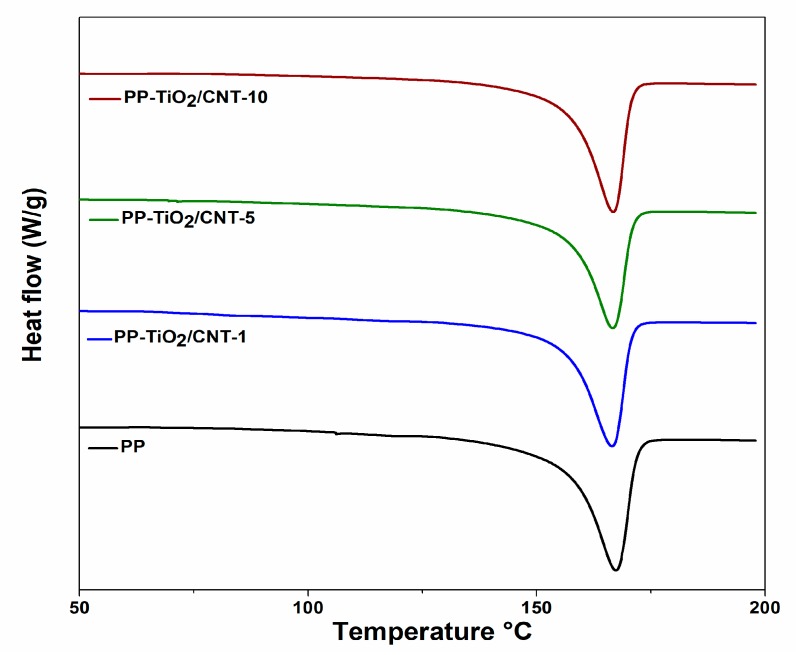
Differential scanning calorimetry (DSC) fusion endotherms of pure polypropylene (PP) and PP-TiO_2_/CNT (carbon nanotubes) (1, 5, 10%).

By DSC analysis, the melting temperature T_m_, crystallization temperature Tc and degree of crystallinity Xc, were derived from endothermic and exothermic peak temperatures. These thermograms are shown in Figure 5 and Figure 6. 

The DSC analyses reported that Tc and Tm increase gradually with the increase of nanoparticles (TiO_2_ and CNT) in the PP, reaching temperatures above 122 °C and 157 °C, respectively.

The crystallization process can be noted in the DSC curves; in Table 4 is the summary of fusion enthalpies, crystallization, and degree of crystallinity calculated with Equation (1), for each of the studied materials. The presence of nanocharges affects PP crystallinity, the fusion peaks are narrow in comparison to peak from neat PP, Xc decreases in the extent the nanocharges content (CNT and TiO_2_) increases, for nanocomposite with greater content of charges Xc decreased 3.38 wt %, and this behavior can be related to the agglomeration of particles. Some reports indicate that formation of nanocomposites with high content of CNT can induce effects of topologic confinement that can eventually result in reduction of nucleation kinetics and crystallization [47,48,49,50].

In 2015, Zhang et al. [51], in their research with PP and CNT nanocomposites, suggest that variation of crystallization temperature is strongly related to different functions that charges have during PP crystallization.

The effect caused by CNT and TiO_2_ nanoparticles in the PP matrix is known. Some reports are contradictory because the physicochemical characteristics of the nanoparticles are not always the same, so the aggregation and dispersion in semicrystalline polymers is different. On the other hand, the final properties of most semicrystalline polymers depend on the microstructures, which are mainly affected by crystallization [52]. The polymer crystallization may be intimately related to the type of nanoparticle and concentration, dispersion state, aspect ratio, crystallization conditions, and so on. [53,54,55,56,57] All these conditions can explain the contradictory results that have been reported; in the PP-TiO_2_/CNT nanocomposites, the presence of two nanocharges type of blend increases the adverse effects.

The results obtained for the PP-TiO_2_/CNT nanocomposites are expected and they coincide with what is reported in literature, where the effect of CNT and TiO_2_ nanoparticles individually has been studied. For example, several reports show that CNT accelerates PP crystallization only at contents less than 0.5 wt % due to strong heterogeneous nucleation effect of the CNTs in the PP. When the concentration increases, the formation of aggregates is favored, and the heterogeneous nucleating efficiency of the individual CNTs is lowered [58].

The PP/TiO_2_ nanocomposites have similar behavior independent of size and shape of nanoparticles, Xc increases with contents of TiO_2_ of 0.5–2 wt %, when the content increases between 3–4 wt %, decreases it, but the value is similar to that shown for PP [22].

In summary, the high CNT content decreases crystallinity, whereas TiO_2_ increases the crystallinity, even at high contents of ∼2%. The nanocomposites of PP/TiO_2_/CNT have a ratio of CNT/TiO_2_ of 2:1. Therefore, the expected effect for CNT on the PP matrix is more predominant. According to Table 4, a decrease was detected in Xc and it decreases in the extent of CNT content increases.

### 3.6. Electron Scan Microscopy

**Figure 7:**
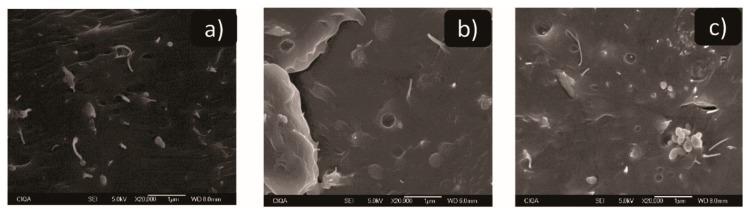
SEM micrographs of nanocomposites. (**a**) polypropylene-carbon-nanotubes (PP-TiO_2_/CNT-1), (**b**) PP-TiO_2_/CNT-5 and (**c**) PP-TiO_2_/CNT-10.

Figure 7 shows SEM micrographs of PP-TiO_2_/CNT-1, PP-TiO_2_/CNT-5, and PP-TiO_2_/CNT-10 nanocomposites. In these SEM images, the presence of CNT and TiO_2_ nanoparticles can be verified. All micrographs exhibited two zones with different nanoparticles agglomeration, in zone 1 (Supplementary Materials) agglomerates can be observed containing both nanoparticles and their contact between them. Also, the amount of agglomerates increases with the increase in the CNT and TiO_2_ content. The zone 2 showed less agglomeration and it was noted that both nanoparticles are embedded within PP matrix (Figure 7a–c). In these zones, it is difficult to define if CNT and TiO_2_ nanoparticles are in contact. In the micrography of PP-TiO_2_/CNT-10 nanocomposite with greater content of nanoparticles (Figure 7c), small agglomerates were detected where there is contact between TiO_2_ nanoparticles. It is worth to mention that processing conditions of nanocomposites causes coalescence between TiO_2_ nanoparticles and in some cases the spherical shape it is not well defined; similar results were previously reported [59]. The coalescence is not exclusive to TiO_2_ NPs. The spherical copper nanoparticles (Cu NPs), with an average size of 21 nm when they are processed to obtain Nylon 6/Cu nanocomposites, increased their size and formed aggregates in form of wire [60]. 

The increase in viscosity caused coalescence of the TiO_2_ NPs, the PP-TiO_2_/CNT-10 had an average particle size of 200 nm, and the nanocomposites with 1 and 5 wt % of NPs had an average particle size of 557 nm and 286 nm, respectively (Supplementary Materials Figures S1–S3).

In some of the micrographs, a series of cavities or voids can be observed in the polymeric matrix. This can be due to the fact that the extrusion process of the particles of greater size is sent against the next material, impacting and forming a track or trail [61].

### 3.7. Electrical Conductivity

The electric resistance of nanocomposites decreases to the extent the nanoparticles content increases; this effect was only significant in the PP-TiO_2_/CNT-10 nanocomposite. With the addition of 10% of nanoparticles, the electrical conductivity increased 6 times with regards to pure polypropylene. Table 5 lists the obtained values of electrical resistance and calculated electrical conductivity. The lack of electrical conductivity in nanocomposites with 1 and 5 wt % can be explained by an inefficient dispersion of nanoparticles, and this coincides with SEM analysis. Another possible explanation can be found in the amount of PP that can be bonded to nanoparticles. The adherence of polymer chains to nanoparticles surface prevents electrons flow, and it is known that thermal treatments can improve the electrical conductivity, due to destruction of crystalline phase of existent polymer in the surface [62]. 

The electrical conductivity of PP/CNT nanocomposites has been deeply studied and it is known that nanocomposites with content of CNT of 10 wt % can have resistivity of up to 10^2^ Ω/sq [63]. 

There are few studies about electrical properties of PP/TiO_2_ nanocomposites. To our knowledge, there is only one study about this, and is reported that with the increase in volume content of titanium-dioxide nanoparticles, the value of dielectric permittivity of nanocomposites also increases, and after some point it starts to decrease. The specific resistance of nanocomposites depends on temperature and it was detected that at 116.9 °C the electrical properties of nanocomposite show significant improvement [64].

### 3.8. Mechanical Properties

The results of mechanical tests are listed in Table 6, where tensile strength, percentage of nominal deformation to rupture, and Young’s modulus values for nanocomposites can be noted. In this analysis, it was observed PP without charge presented a tensile strength of 23.93 MPa, while the PP-TiO_2_/CNT nanocomposites showed low values, which decreases in the extent the nanoparticles content is increased. These values coincide with the degree of crystallinity (Table 4); in consequence, the decrease of rigidity of matrix of PP is favored. 

For the nominal percentage at the breaking the observed behavior is similar, such effect is due to the fact that nanotubes particles obstruct the movement of PP chains, reducing rigidity. Young’s modulus of PP-TiO_2_/CNT-10 nanocomposite showed an increase of 18.3% with regards PP without charges. This was the only case where it was observed that nanoparticles cause higher rigidity and hardness in the PP matrix. It has been reported that adding CNT in polymers will strengthen material, if there is an efficient dispersion [65]

The decrease of Young’s modulus when increasing charges content is explained by inefficient dispersion of charges, since the agglomerates present in poorly dispersed composite cause cracks to initiate and easily propagate in polymeric matrix [66]. As noted previously, the TiO_2_ nanoparticles tend to coalesce, increasing their size and changing shape. This process seems to be more significant when charges concentrates are lower (1 and 5%). This behavior can be attributed to the increase of viscosity with the nanoparticles’ concentration. For low viscosity, the TiO_2_ NPs are able to move in the matrix and aggregates, whereas at high concentrations the viscosity is higher and the aggregation is limited by slightly mobility of the nanoparticles.

### 3.9. Calorimetric Cone

To analyze combustion processes, in real time assessment of nanocomposites the cone calorimetry method was used. Heat release rate (HRR) and its maximum value (PHRR) were some of the parameters obtained in this study.

**Figure 8:**
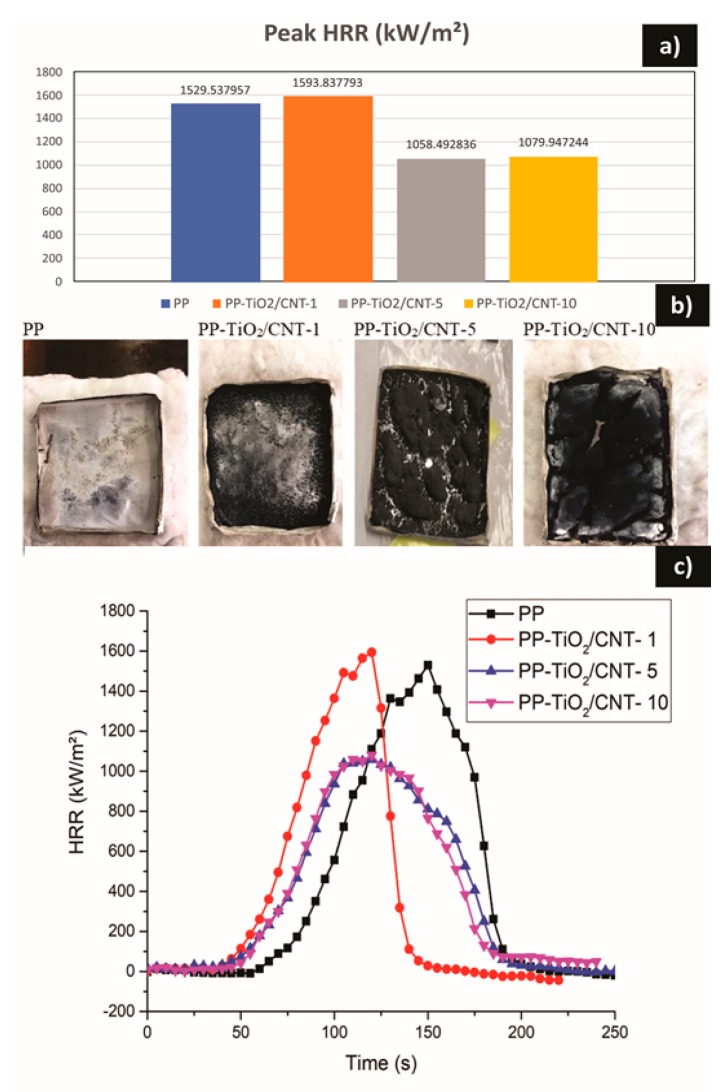
Calorimetric measurements (**a**) Comparison of the peaks of the heat release rate (HRR), (**b**) Photographs of residual material after the cone calorimetric test (**c**) HRR curves for the results.

The heat release rate curves of the PP-TiO_2_-CNT nanocomposite are shown in Figure 8. These results showed essentially a similar behavior, the maximum value of heat release rate (PHRR) for PP without charge and PP-TiO_2_/CNT-1 nanocomposite were of 1529.53 and 1593.83 kW/m² respectively, which means that there is no difference between pure PP and nanocomposite with low load percentages (1%). In contrast, for PP-TiO_2_/CNT-5 and TiO_2_/CNT-10 nanocomposites, a decrease was noted in PHHR and total heat release rate (THR) values, which indicates that materials have less flame propagation and better resistance to fire. The minimum values of PHHR that could be obtained were of 1058.49 kW/m² for PP-TiO_2_/CNT-5 and of 1079.94 kW/m² for PP-TiO_2_/CNT-10.

Table 7 lists data of calorimetric cone, where it was noted that the total heat release rate (THR) reduced when increasing the particles content to 5 and 10% wt. Also, there are listed the wt % of residues obtained after flame-retardant assessment. The number of residues increases dramatically when the nanoparticles content increases. For PP-TiO_2_/CNT-10 nanocomposite, the obtained residues were 91.9%. In Figure 8b) the residues appearance can be observed. For the case of PP-TiO_2_/CNT-1, it was noted that there was a small amount of scattered dust, while for PP-TiO_2_/CNT-5 y TiO_2_/CNT-10 a semi-continuous phase that suggests a good distribution of nanocharges in polypropylene matrix was noted. In addition, during the ignition a network forms between nanoparticles and degradation product of PP, and the new materials can have enough resistance to flame in order to avoid complete ignition. A similar explanation was obtained by studying the flammability properties of the PP/zeolite/CNT nanocomposite, where it was proposed to form a protective layer with a continuous network structure that provides the flame-retardant characteristic of polymeric nanocomposites [67].

Results coincide with other studies, for example, PP/CNT nanocomposites are considered to be flame-retardant materials and even more effective than PP/clay nanocomposites. The combination of TiO_2_ and CNT to obtain anti-flame additives can be a good idea, because TiO_2_ has a high decomposition temperature (700 °C–800 °C) and an oxygen index of 29 and has also been widely used as an anti-flame additive alone or in combination with other additives [5]. Despite the above, its usage, like flame-retardant in polyamides, has been technically questionable [68]. 

In our study, the CNT and TiO_2_ blends lead to obtaining nanocomposites with acceptable flame-retardant properties, but more detailed studies can be required to optimize formulation and to explain its possible mechanism.

## 4. Conclusions

The PP-TiO_2_/CNT nanocomposites was obtained by the melt mixing method using a mixture of nanoparticles TiO_2_ and CNT, with contents of 1, 5, and 10 wt %. The thermal stability of the nanocomposites increased when increasing the content of the NPs, for example, for PP-TiO_2_/CNT-10, its maximum degradation temperature increased 16 °C with respect to the pure PP. In addition, the thermo-oxidative stability of the material was improved and confirmed by the lack of signals of the carbonyl group in FTIR spectrum. The degree of crystallinity decreased with a high content of NPs. This effect was also reflected in a slight decrease in mechanical properties, and only an in increase in the Young’s modulus of 10% to PP-TiO_2_/CNT-10 was observed.

The electrical conductivity of PP-TiO_2_/CNT-10 nanocomposite was improved by eight orders of magnitude with respect to the pure PP; nanocomposites with low content do not have significant changes.

The melt flow index (MFI) of the nanocomposites decreased with the number of NPs, and the nanocomposites with 1, 5, and 10 wt % gave an MFI of 0.56, 0.40, and 0.24 (g/10 min) respectively. The conditions of the melt processing and the increase in the viscosity caused coalescence of the TiO_2_ NPs. This conducted to different average particle size in each nanocomposite.

Finally, the effect of flame retardancy was confirmed by a significant decrease of the peak HRR in the nanocomposites PP-TiO_2_/CNT-5 and PP-TiO_2_/CNT-10, and besides this, PP-TiO_2_/CNT-10 presented a content of residual carbon of 91.9% after ignition.

## Figures and Tables

**Table 1:** Compounding formulations polypropylene-carbon-nanotubes (PP-TiO_2_/CNT-X).

Sample	CNT + TiO_2_ (wt %)	PP (g)	TiO_2_/CNT (g)
PP	0	200	0
PP-TiO_2_/CNT-1	1	198	2
PP-TiO_2_/CNT-5	5	190	10
PP-TiO_2_/CNT-10	10	180	20

**Table 2:** Flow index of the samples analyzed.

Sample	MFI (g/10 min)
PP	0.76
PP-TiO_2_/CNT-1	0.56
PP-TiO_2_/CNT-5	0.40
PP-TiO_2_/CNT-10	0.24

**Table 3:** Thermal properties of nanocomposite of polypropylene-carbon-nanotubes (PP-TiO_2_/CNT).

Sample	T_5%_ (°C)	T_50%_ (°C)	T_max_ (°C)	Residue at 550 °C (%)
PP	417	454	459	0
PP-TiO_2_/CNT-1	435	460	462	1.00
PP-TiO_2_/CNT-5	448	469	472.	4.92
PP-TiO_2_/CNT-10	454	474	475	8.02

**Table 4:** Differential scanning calorimetry (DSC) date for polypropylene (PP) and nanocomposites PP-TiO_2_/CNT (carbon nanotubes) (1, 5 y 10%).

Nanocomposite	T_m_ (°C)	Enthalpy of Fusion (J/g)	Enthalpy of Crystallization (J/g)	*X_c_* (*%*)
PP	156.93	95.82	93.72	45.84
PP-TiO_2_/CNT-1	156.16	93.45	95.35	44.71
PP-TiO_2_/CNT-5	157.14	91.06	92.82	43.56
PP-TiO_2_/CNT-10	157.07	88.75	93.63	42.46

**Table 5:** Electrical properties of polypropylene (PP) and PP-TiO_2_/CNT (carbon-nanotubes) nanocomposites.

Sample	Surface Resistance Ω/sq	Volumetric Resistance Ω cm	Electric Conductivity S/m
PP	2.35 × 10^13^	1 × 10^18^	1 × 10^−18^
PP-TiO_2_/CNT-1	6 × 10 ^16^	3 × 10^17^	3.0 × 10^−18^
PP-TiO_2_/CNT-5	3 × 10^12^	5 × 10^16^	2.0 × 10^−17^
PP-TiO_2_/CNT-10	6.5 × 10^9^	7.2 × 10^9^	1.4 × 10^−10^

**Table 6:** Tensile properties of polypropylene (PP) and PP-TiO_2_/CNT (carbon nanotubes) nanocomposites.

Sample	Xc	Tensile Strength (MPa)	Nominal Strain at Break (%)	Young’s Modulus (MPa)
PP	56.36	23.93 ± 0.12	53.37	971.57 ± 29.1
PP-TiO_2_/CNT-1	54.97	23.93 ± 0.19	45.78	894.98 ± 27.1
PP-TiO_2_/CNT-5	53.56	23.32 ± 0.08	33.24	961.28 ± 31.0
PP-TiO_2_/CNT-10	52.20	22.75 ± 0.37	22.28	1077.44 ± 26.2

**Table 7:** Data of the calorimetric cone test of the samples analyzed.

Sample	Peak HRR (kW/m²)	THR (MJ/m²)	Residue (%)
PP	1529.53	66.42	0.09
PP-TiO_2_/CNT-1	1593.83	65.91	34.3
PP-TiO_2_/CNT-5	1058.49	43.49	88.4
PP-TiO_2_/CNT-10	1079.94	44.06	91.9

## Supplementary Materials

The following are available online at https://www.mdpi.com/2073-4360/11/7/1204/s1. Figure S1. Morphology nanocomposite PP-TiO_2_/CNT-1 analyzed by SEM, histogram of TiO_2_ particle. Figure S2. Morphology nanocomposite PP-TiO_2_/CNT-5 analyzed by SEM, histogram of TiO_2_ particle. Figure S3. Morphology nanocomposite PP-TiO_2_/CNT-10 analyzed by SEM, histogram of TiO_2_ particle.
