# Supplementary material for: Melt-Mixed Thermoplastic Nanocomposite Containing Carbon Nanotubes and Titanium Dioxide for Flame Retardancy Applications
Source: Polymers (Basel). 2019 Jul 19;11(7):1204. doi: 10.3390/polym11071204 (PMC6680381; doi:10.3390/polym11071204)
Supplement: Supplementary file 1 [file polymers-11-01204-s001.pdf]

polymers

Supplementary Materials: The following are available online at www.mdpi.com/2073-4360/11/7/1204/s1.

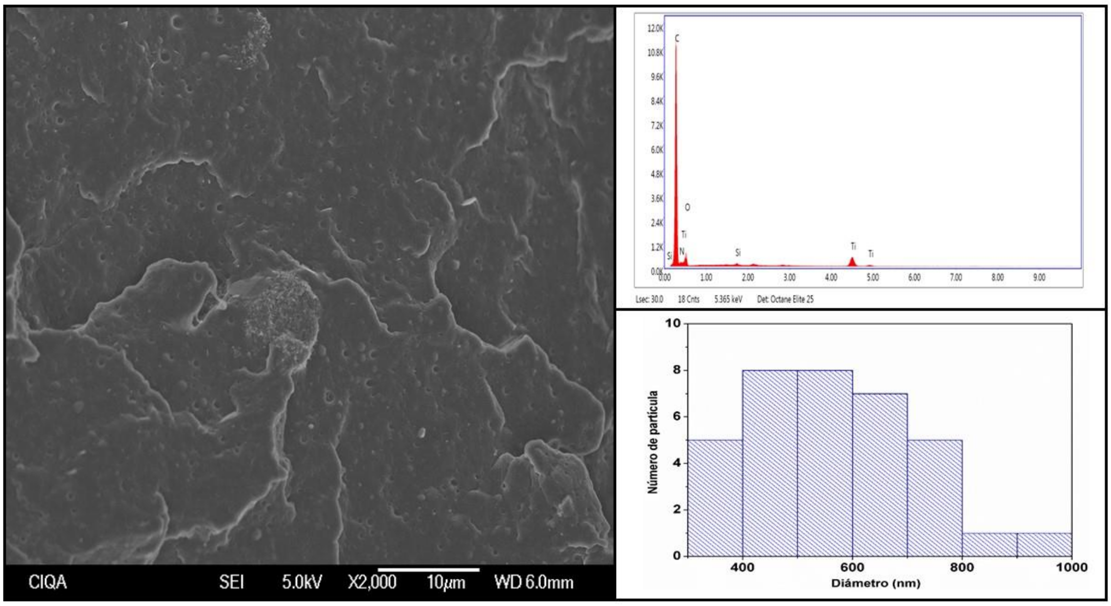

Figure S1. Morphology nanocomposite PP-TiO_2/CNT-1 analyzed by SEM, histogram of TiO_2 particle.

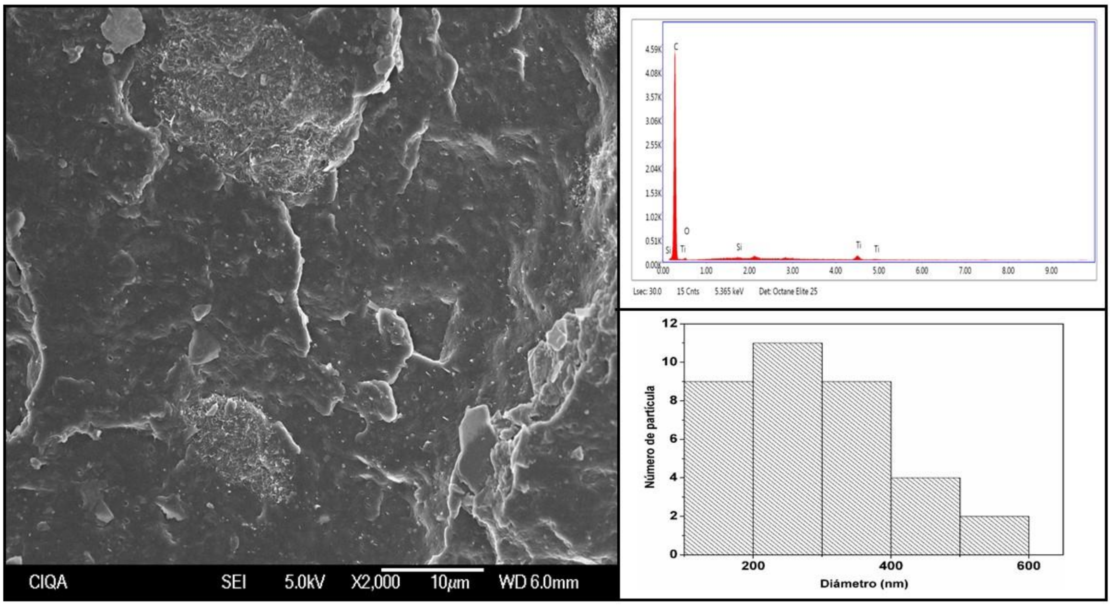

Figure S2. Morphology nanocomposite PP-TiO_2/CNT-5 analyzed by SEM, histogram of TiO_2 particle.

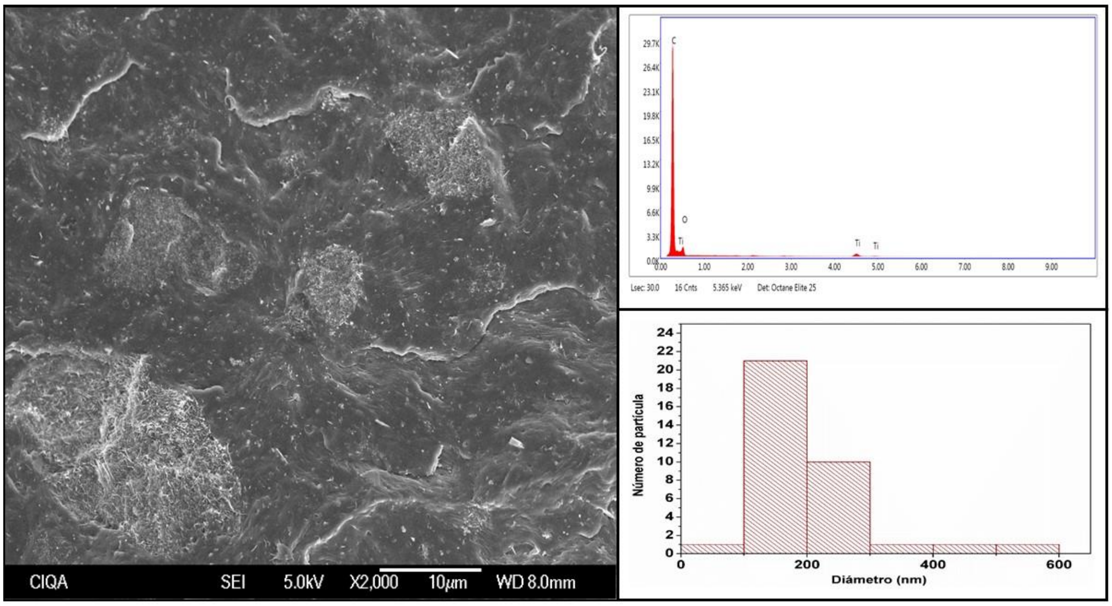

Figure S3. Morphology nanocomposite PP-TiO_2/CNT-10 analyzed by SEM, histogram of TiO_2 particle.
